# A Review of Studies Supporting Metaphorical Embodiment

**DOI:** 10.3390/bs13070585

**Published:** 2023-07-13

**Authors:** Omid Khatin-Zadeh, Danyal Farsani, Jiehui Hu, Zahra Eskandari, Yanjiao Zhu, Hassan Banaruee

**Affiliations:** 1 School of Foreign Languages, University of Electronic Science and Technology of China, Chengdu 610054, China; khatinzadeh.omid@yahoo.com (O.K.-Z.); jerrywho@163.com (J.H.); zhuyan.jiao@hotmail.com (Y.Z.); 2 Department of Teacher Education, Norwegian University of Science and Technology, 7491 Trondheim, Norway; 3 Department of English, Chabahar Maritime University, Chabahar 99717-56499, Iran; eskandari62@gmail.com; 4 Department of English, American, and Celtic Studies, University of Bonn, 53115 Bonn, Germany; hassan.banaruee@uni-bonn.de

**Keywords:** metaphorical embodiment, sensorimotor systems, sensorimotor strength, simulation

## Abstract

This paper presents a review of studies that have provided evidence supporting metaphorical embodiment. These studies are divided into three categories of behavioral, neuroimaging, and corpus studies. After summing up the findings of these studies, it is concluded that metaphorical embodiment is supported by these three lines of research. This is followed by a review of a number of studies that have measured sensorimotor and action effector strengths of various concepts. Then, the idea of sensorimotor and action effector strength of concepts is linked to metaphorical embodiment to present the main idea of the paper. Based on the findings of studies that have measured sensorimotor and action effector strengths of concepts, it is suggested that the degree of involvement of sensorimotor systems in mental simulation of metaphoric actions may not be at the same level in all metaphors. It depends on the sensorimotor strength of the base of the metaphor in various modalities. If the base of a metaphor has a high degree of perceptual strength in a certain modality, that modality plays the most important role in the processing of that metaphor, while other modalities take less important roles. In other words, depending on the sensorimotor strengths of the base of a metaphor in various modalities, those modalities have various levels of importance in the processing of that metaphor. If the base of the metaphor is weak in all modalities, modal resources can come into play to process that metaphor.

## 1. Introduction

Embodied cognition and theories of embodiment have been the subject of a large volume of interdisciplinary research in recent years. Embodied cognition refers to reactivation of previously experienced sensorimotor processes when people think or talk about objects and concepts [1]. Importantly, the sensorimotor processes experienced during actual perception of an object can be reactivated and re-experienced when people talk about it, think about it, or imagine it, even in the absence of that object [1,2,3,4,5]. In other words, sensorimotor processes associated with real perception of an object can be reactivated by using a word that refers to that object or just thinking about that object without using a word. The emergence of the conceptual metaphor theory [1], which originated from embodiment theories, was a milestone and the starting point for a new line of theoretical and empirical research in metaphor studies. This theory inspired researchers in various fields, such as cognitive science, cognitive linguistics, and cognitive psychology. In the past four decades, a continuously rising number of research projects has provided evidence that supports this theory or seems to question some of its assumptions. According to this theory, metaphor is used to describe and understand one concept (target of the metaphor) in terms of another concept (base of the metaphor), usually an abstract (or an unfamiliar) concept in terms of a concrete (or familiar) concept [1]. For example, in the conceptual metaphor LOVE IS A JOURNEY, the abstract concept of *love* (target of the metaphor) is partially structured, understood, and talked about in terms of *journey* (base of the metaphor). Although *love* and *journey* have very different literal meanings and semantic associations when used in their literal senses, *love* can be metaphorically understood in terms of a *journey*. Lakoff and Johnson [1] argue that a small set of conceptual metaphors underlies a much larger number of metaphorical expressions in the language. In other words, a single conceptual metaphor can be the source of a large number of metaphors that are realized in a variety of linguistic forms in the language. For example, the metaphoric expressions *I won the argument*, *my argument was attacked by the members of the party*, and *I demolished her argument* are realizations of the conceptual metaphor ARGUMENT IS A WAR. Although these metaphoric sentences differ in their words and their linguistic structures, they are the same at a conceptual level. These three metaphors and a much larger number of other metaphorical expressions in a variety of forms originate from a single conceptual or higher-order metaphor. Gibbs [2] argues that hearing a metaphoric sentence activates the conceptual metaphor that underlies that metaphoric sentence. This means that hearing each realization of a given conceptual metaphor activates its corresponding higher-level conceptual metaphor.

Metaphorical embodiment is an influential view that builds on the conceptual metaphor theory. This view was initially proposed by Gallese and Lakoff [3] and later was developed and modified by some subsequent works (e.g., [4,5]). According to this view, the same neural networks and brain structures involved in processing the base of a metaphor are actively recruited to process the target of the metaphor [3]. For example, in the metaphor *I grasped the idea*, the process of understanding an idea (target of the metaphor) is described in terms of grasping a physical object (base of the metaphor). Therefore, the motor system, which guides and controls body actions such as grasping, plays an active role in the processing of this metaphor. The main idea of metaphorical embodiment is that the same neural networks and brain structures that are recruited or activated during performing the action of grasping a physical object are employed or activated to process this metaphor. In other words, the same sensorimotor networks that are employed to do the action of grasping are recruited to understand the target of the metaphor (understanding an idea) in terms of its base (grasping a physical object). From the perspective of metaphorical embodiment, when a metaphor is processed, its target is embodied in terms of its base. Since this theory makes strong claims about the process of embodiment, it has been called one of the strong versions of embodiment [6,7,8,9,10,11,12,13].

Like the conceptual metaphor theory, metaphorical embodiment has been the subject of a large volume of theoretical and empirical works in recent years. Generally, works on metaphorical embodiment can be categorized into behavioral, neuroimaging, and corpus studies. In this paper, behavioral studies that have supported metaphorical embodiment are divided into four subcategories: (1) action-sentence compatibility studies; (2) eye-tracking studies; (3) hand-prime studies; (4) gesture-in-learning studies. Neuroimaging studies that have supported metaphorical embodiment are classified into two subcategories: (1) studies on sensorimotor systems activation during metaphor processing; (2) studies on sensorimotor activation during mental imagery. After reviewing several corpus studies that have supported metaphorical embodiment, an organized summary of evidence for the key assumptions of metaphorical embodiment is presented. Finally, a number of studies that have measured sensorimotor and action effector strengths of various concepts are reviewed, and the idea of sensorimotor strength of concepts is linked to metaphorical embodiment to present the main idea of the paper. Based on the findings of studies that have measured sensorimotor and action effector strengths of concepts, it is suggested that the degree of involvement of sensorimotor systems in mental simulation of metaphoric actions may not be at the same level in all metaphors. It depends on the sensorimotor strength of the base of the metaphor in various modalities.

## 2. Behavioral Studies on Metaphorical Processing

### 2.1. Action-Sentence Compatibility Studies

In this category of studies, the compatibility of a body action with the metaphoric meaning of a verb is examined. Studies on action-sentence compatibility effect have been conducted on both literal and metaphoric sentences. Here, only studies that have investigated metaphoric action-sentence compatibility effect are reviewed, as this type of work is relevant to the content of this review paper. In the past two decades, results of many action-sentence compatibility studies have suggested that when a body action is compatible with the literal or metaphoric meaning of a verb, it is easier for people to make a judgment on the sensibility of the metaphoric verb by that body action, e.g., [14,15,16,17,18,19,20,21]. This can be taken as an evidence that the meaning of metaphoric verb is simulated in the mind when a sentence that includes that verb is processed. In one of the most well-known action-sentence compatibility studies [22], participants made judgments on the sensibility of a set of sentences that described the directed transfer of abstract entities, such as, ‘The policeman radioed the message to you’, and ’You radioed the message to the policeman’. The participants made such judgments by moving their hands toward or away from their bodies. The results showed that when a sentence referred to a metaphoric action in one direction, the participants had difficulty in making a sensibility judgment by making a response in the opposite direction. This has been called the action-sentence compatibility effect. For example, it was easier for the participants to give a positive answer to the sentence *you radioed the message to the policeman* by a movement away from the body than by a movement toward the body. This suggests that the action of sending a message from the sender to the receiver was simulated when the participants were processing the sentence. In other words, the action of sending a message was metaphorically simulated in terms of sending a concrete object from the sender to the receiver. This is why the participants were faced with difficulty in making a sensibility judgment that required an action in the opposite direction. In an extension of this study, Borreggine and Kaschak [23] used the same sentences that described the metaphorical transfer of abstract entities and found that the action-sentence compatibility effect arose only when participants were given the opportunity to plan their motor response while they were processing the sentence. The results of this study again suggested that the metaphorical action of transferring abstract entities is mentally simulated or embodied when such sentences are processed.

In a recent study [24], the action-sentence compatibility effect was examined for a set of abstract verbs with low motor associations and a set of novel words. In the learning phase of the first experiment, motor features of a set of abstract verbs with low motor features were strengthened. In the learning phase of the second experiment, motor features of a set of novel words were strengthened. After adding the motor experiences in the learning phases, a significant degree of action-sentence compatibility effect was observed in the test phases of experiments. The interesting point about the findings of this study was that when some motor experiences were associated with even novel words, action-sentence compatibility effect was observed. The results of all such studies suggest that simulation of actions takes place not only for literal verbs but also for metaphoric verbs, which supports they key idea of metaphorical embodiment.

One study reported that metaphoric action-sentence compatibility effect has an impact on discourse comprehension [25]. Participants of this study read a text describing an individual making metaphoric forward movements. In the first experiment, participants’ body movements were manipulated to be compatible or incompatible with metaphoric actions. In the second experiment, participants’ body postures were manipulated to be compatible or incompatible with the metaphoric actions. The results of both experiments showed that compatibility of body movement and body posture with metaphoric action enhanced participants’ comprehension of discourse. This means that compatibility of body movement or even body posture with the metaphoric action described in a discourse affects global understanding of the discourse, while incompatibility of body movement or body posture with the metaphoric action described in the discourse can negatively affect discourse comprehension. The results of all studies reviewed in this section suggest that performing an action that is not compatible with a metaphoric action and maybe even having the intention to perform an action that is not compatible with a metaphoric action disrupts the process of sentence comprehension and discourse comprehension.

### 2.2. Eye-Tracking Studies

In this category of studies, traces of eye movements during metaphor processing are examined. The results of some of these studies have shown that when a metaphoric or a literal sentence is processed, the direction of eye movement is compatible with the direction of the movement that is described by the metaphoric or literal verb, e.g., [26,27,28,29,30,31,32,33,34]. For example, in the metaphoric sentence *he rose up in the hierarchy of the company*, promotion is metaphorically described in terms of an upward movement. The literal sentence *the sun rose up* describes the upward movement of the sun. The results of many eye-tracking studies have shown that processing such metaphoric and literal sentences is accompanied by upward eye movement. This has been taken as evidence suggesting that metaphoric and literal verbs are mentally simulated and are realized in upward eye movements. Here, we limit our review of eye-tracking studies to metaphor processing research.

Richardson and Matlock [35] examined eye movements of a group of participants when they were looking at pictures that were being described by metaphoric fictive motion sentences (e.g., the road goes through the desert). In some cases, the contextual information indicated that the terrain was difficult to pass, while in other cases the context indicated that the terrain was easy to pass. The results showed that the length of time of inspections and eye movements scanning along the path increased during fictive motion descriptions that had been initially described as difficult (the desert is hilly) as compared to easy (the desert is flat). Interestingly, the difference in inspection times and eye movements was not observed when the pictures were described without fictive motions. This suggests that fictive motions are mentally simulated and these mental simulations are realized in eye movements. Castaño and Carrol [36] examined patterns of eye movements of a group of participants during processing literal sentences that described actual physical motions (e.g., the curtain is rising) and metaphoric sentences that described changes in quantity or emotional states in terms of motion (e.g., prices are rising). The results of this study showed that eye movements were mostly compatible with the direction implied by the verb, regardless of whether the verb had been used in a literal or metaphorical sense. Similar to the findings of the previous study, the results of this study again suggested that metaphoric movements are simulated in terms of patterns of eye movement during comprehending metaphoric sentences.

Mishra and Singh [37] examined fixational eye movements of a group of participants looking at images that were described by either fictive motion sentences or nonfictive motion sentences. The results showed significant gaze durations and number of fixations during comprehension of fictive motion sentences compared to nonfictive motion sentences. This suggests that the mode of visual processing of a picture described by a fictive motion sentence is somehow different from the mode of visual processing of the same picture described by a nonfictive motion sentence. In a related study, Singh and Mishra [38] found that comprehenders gazed for a longer period of time at visual scenes when hearing metaphoric fictive motion sentences compared to literal sentences. This indicates that when a static image is metaphorically/fictively described in terms of a motion event, that motion is mentally simulated and realized in the patterns of eye movements.

All these studies suggest that when a static concept is metaphorically described in terms of a motion event (e.g., time is a moving) or in terms of a fictive motion (e.g., the road passes through the jungle), the motion is mentally simulated. This mental simulation may be physically realized or embodied in various parts of the body, such as hand gestures or eye movements. However, there is an important difference between the embodiment of metaphoric actions in eye movements and other parts of the body such as hand gestures. While hand gestures can have an active communicative role, eye movements do not have such a function. People may use hand gestures to communicate more information and emphasize something, but eye movement cannot have such a communicative function. Patterns of eye movement during processing metaphoric sentences can be the reflection of a mental simulation. In other words, they are the physical realizations or the embodiment of a fully mental simulation. Therefore, patterns of eye movements can be taken as strong evidence that supports metaphorical embodiment.

### 2.3. Hand-Prime Studies

In this category of studies, the impact of a hand gesture on the understanding of a subsequent metaphor is investigated. The hand gesture, which is produced or imagined by the comprehenders or is visually presented to them, functions as a prime. In the first major study of this category, Wilson and Gibbs [39] conducted an experiment to find out how producing a real body movement or imagining a body movement affects the process of understanding a subsequent metaphor. In each item of one experiment, a metaphor that described a concept in terms of a metaphoric action was used. Participants produced a body movement that depicted the metaphoric action and then immediately read the metaphor. For example, they produced a pushing movement and then read the metaphorical phrase *push an argument*. In each item of another experiment, the participants imagined the metaphoric action and then immediately read the metaphor. The results of these experiments showed that performing or even imagining a body movement that depicted the metaphoric action of a subsequent metaphor could enhance the process of understanding the metaphor. This suggests that a metaphoric action such as pushing is simulated or embodied when the metaphorical phrase *push an argument* is processed. In other words, the motor system, which guides the action of pushing, plays an active role in grounding the target of this metaphor in the metaphoric action. Therefore, when the motor system is activated by performing or imagining a pushing movement, it is more prepared to contribute to the processing of this metaphorical phrase. In fact, the motor system, as one of the cognitive resources involved in the processing of this metaphorical phrase, facilitates and enhances the process of comprehending this metaphorical phrase.

If a gesture that depicts the metaphoric action of a metaphor can facilitate the process of understanding the metaphor, it can be predicted that a gesture that is incongruent with the metaphoric action can disrupt the process of understanding the metaphor. This question was investigated by two recent priming studies on metaphorical embodiment. In one study [40], different groups of participants made sensibility judgments on a set of 20 metaphors. One group made judgments in congruent gesture-prime conditions, while the other group made judgments in incongruent gesture-prime conditions. Another group made judgments in no-prime conditions. In the congruent gesture-prime conditions, each metaphor was preceded by a gesture that depicted the schema of the subsequent metaphor. That is, it depicted a metaphoric action or something related to the base of the subsequent metaphor. In incongruent gesture-prime conditions, each metaphor was preceded by a gesture that was incongruent with the schema of the subsequent metaphor. The results showed that in congruent gesture-prime conditions, a higher proportion of sentences were judged to be sensible and sensibility judgments were made faster compared to those in no-prime and incongruent gesture-prime conditions. Since metaphor schema is a depiction of the metaphoric action or depicts something that is related to the metaphoric action, it can be concluded that the metaphoric action or schema of a metaphor is simulated in the mind of the comprehender during metaphor processing. Therefore, when the schema of a metaphor is presented to the comprehenders just before processing a metaphor, their comprehension of the metaphor is facilitated. In another related study, Khatin-Zadeh [41] examined three groups of participants’ interpretations of a set of metaphors in three different conditions: congruent gesture-prime conditions, opposite gesture-prime conditions, and no-prime conditions. In congruent gesture-prime conditions, the written version of each metaphor followed a gesture that was congruent with the gestural representation of the metaphor schema. In opposite gesture-prime conditions, the written version of each metaphor followed a gesture that was incongruent with the gestural representation of the metaphor schema. The results showed that the best interpretations were given in the congruent gesture-prime conditions, while the weakest interpretations were given in opposite gesture-prime conditions.

### 2.4. Gesture-in-Learning Studies

The role of gesture in enhancing the process of learning has been demonstrated by a large number of studies, e.g., [42,43,44,45,46,47,48,49]. In these studies, the contributions of iconic and metaphoric gestures have been at the focus of research. Iconic gestures present an illustration of the shapes of objects they refer to and have a direct relationship with the semantic content of their referents [50], while metaphoric gestures present an illustration of the base of a metaphor and have an indirect relationship with the semantic content of the target of the metaphor. This section of the review focuses on studies that have investigated the role of metaphoric gestures in enhancing the process of learning and emphasizes that this function of metaphoric gesture supports metaphorical embodiment. In various branches of science, many concepts are metaphorically described in terms of easy-to-understand representations. For example, in mathematics, the arithmetic operations of addition and subtraction are metaphorically described in terms of rightward movements (or right space) and leftward movements (or left space) on an axis, respectively, e.g., [51,52,53]. These rightward and leftward movements can be illustrated by metaphoric gestures. It has been demonstrated these metaphoric gestures can play an active role in enhancing the process of learning these arithmetic operations, e.g., [54].

Results of these studies can also be taken as a support for metaphorical embodiment, because they suggest that abstract concepts can be mentally simulated, reflected in gestures, and grounded into concrete environment through the mediation of sensorimotor systems. In fact, metaphoric gestures are reflections of mental processes. Gestures are concrete realizations of these mental processes. Therefore, it can be said that the contribution of metaphoric gestures to learning processes can be taken as supporting evidence for metaphorical embodiment, because it supports the idea that metaphoric gestures are physical realizations of mental processes that take place through the active involvement of sensorimotor systems. Hostetter and Alibali [55,56] propose the gesture-as-simulated-action theory and argue that using iconic gestures along with literal language and using metaphoric gestures along with metaphoric language show that concepts and actions are simulated. Importantly, this happens for both literal and metaphoric language. For example, a pushing gesture that co-occurs with the literal sentence *push the table* is an iconic gesture. Using this iconic gesture shows that the action described by this literal sentence is mentally simulated and reflected in co-speech gestures. Furthermore, a pushing gesture that co-occurs with the metaphoric sentence *push the idea* is a metaphoric gesture. Using this metaphoric gesture indicates that the metaphoric action described by this metaphoric sentence is also mentally simulated and reflected in the accompanying gestures. In other words, using co-speech metaphoric gestures, which is very common in daily language, is evidence that even metaphoric actions are mentally simulated and embodied in co-speech gestures. In fact, metaphorical embodiment is supported by the highly common phenomenon of using co-speech metaphoric gestures with metaphoric language.

## 3. Neuroimaging Studies on Metaphorical Embodiment

### 3.1. Studies on Sensorimotor Systems Activation during Metaphor Processing

Findings of a range of neuroimaging studies have suggested that sensorimotor systems play an active role in the processing of metaphorical expressions, e.g., [57,58,59,60,61,62]. Some of these studies have provided evidence suggesting that the same areas of brain involved in the processing of the base of a metaphor (often a concrete concept) are activated to process the target of the metaphor (often an abstract concept) in terms of the base, supporting one of the key ideas of metaphorical embodiment, e.g., [63,64,65]. In one of these studies, Boulenger, Hauk, and Pulvermüller [66] examined sensorimotor activations in the arm and leg regions of the brain when a set of metaphors that described concepts in terms of arm or leg actions were being processed. They found that when the metaphor *she grasped the idea* was being processed, a noticeable activation in the arm region of the sensorimotor system took place. Furthermore, they observed a significant activation in the leg region of the sensorimotor systems during processing the metaphor *she kicked the habit*. Since the actions of grasping and kicking are metaphoric, results of this study suggest that targets of these metaphors (understanding an idea and quitting a habit) are simulated and embodied in terms of arm and leg actions. Therefore, the same sensorimotor regions involved in performing these body actions are activated to process these metaphors. The results of this study were confirmed by the findings of an MEG study conducted by Boulenger, Shtyrov, and Pulvermüller [67]. They found that processing a metaphoric action like *kick the habit* was modulated by anterior frontotemporal activity. Therefore, they concluded that the meaning of this metaphorical action is reflected by somatotopic activation of precentral motor systems.

Another study [68] made a comparison between sensorimotor activations during processing abstract sentences, literal action sentences, apt but non-idiomatic action metaphors, and action idiomatic sentences. The results of this study showed an increasing trend of sensorimotor activation from abstract to idiomatic to metaphoric to literal sentences. Although the degree of activation of the sensorimotor system was weaker in the processing of action metaphors than literal action sentences, a significant degree of activation was observed in the processing of action metaphors. Such results suggest that the sensorimotor system plays at least a partial role in the processing of metaphoric actions. Yang and Shu [69] conducted meta-analyses of fMRI findings to examine the role of the motor system in the processing of fictive motions, metaphoric actions, and idiomatic actions. The results showed that processing fictive motion sentences involved activation in the right parahippocampal gyrus, an area important for the processing of spatial relations. Also, during processing metaphoric actions, the left precentral gyrus was strongly activated. Based on these results, researchers of this study suggest that there is a link between metaphoric and literal meanings. This means that sensorimotor systems are engaged not only in the processing of literal language and concrete concepts but also in the metaphorical processing of abstract concepts that are described in terms of concrete concepts.

### 3.2. Studies on Sensorimotor Activation during Mental Imagery

A range of neuroimaging studies on visual imagery has provided evidence suggesting that a real motor event and mental simulation of that motor event share the same brain structures and involve activation in the same areas of sensorimotor systems, e.g., [70,71,72,73,74]. The results of these studies have suggested that various content-dependent areas in the ventral visual system are engaged during visual imagery and also visual perception. It has been shown that neural patterns within these regions are similar during imagery and perception [75,76]. This means that visual perception and visuospatial memory use the same neural networks [77].

The key point about the findings of these studies is that mental imagery of objects/events and the activation of visual system can take place in the absence of objects/events that are simulated. If these simulations can take place in the absence of objects/events and without using language (for example, visual recalling of objects/events), they can also take place when language is used to talk about them. In such cases, language can play the role of a medium for activating mental imageries. A mental imagery can be activated by recalling it without talking about it; it can also be activated even more vividly by the support of language, as language can be used to provide and activate more details about the object/event that is simulated. In fact, language can serve as a tool to activate some details that may not be activated in the absence of language use. Importantly, the activation of a mental imagery can take place even by metaphoric language. For example, when the metaphorical phrases *grasp an idea* and *push an argument* are used, the words can activate visual imageries of these metaphoric actions. In this way, the sensorimotor systems that are involved in performing or even observing these actions are activated to simulate or embody these metaphoric actions. Therefore, findings of the studies that have found that visual mental imagery and real visual perception share the same sensory and brain structures have an important implication for metaphorical embodiment. Such findings suggest that sensorimotor systems that are engaged during performing/perceiving a real action can also be engaged when that action is metaphorically used to describe a concept.

## 4. Corpus Studies on Metaphorical Embodiment

In addition to behavioral and neuroimaging studies, some corpus studies have also provided evidence that supports metaphorical embodiment [78,79]. In one of these studies, Johansson-Falk and Gibbs [80] conducted a corpus study on literal and metaphorical uses of *path* and *road*. The results showed that in the literal sentences, the word *path* had been used to talk about varied difficulties in movement and choices between alternatives path of movement. On the other hand, *road* had not been used in this way. Interestingly, the same differences were observed in the metaphorical uses of these words. The results showed that in the metaphorical use of the word *path*, more emphasis was on varied difficulties along the metaphorical path (e.g., their path to a winning was obstructed by an excellent performance from India) and choices between alternatives of action. These were not observed in the metaphorical use of the word *road*. Johansson-Falk and Gibbs [80] concluded that people mentally simulate experiences in journeys along paths and roads and apply them in metaphorical journeys along paths and roads. This is also the case with metaphoric actions. Kövecses [81] reviews a number of corpus studies on universal metaphors such as ANGER IS HEAT across various languages. He argues that the metonymic relationship between the emotional state of anger and the production of heat is one of the reasons behind using this metaphor [82], as behavioral responses to emotional states can function as metonymies in emotion concepts [83,84,85,86,87]. This could mean that when people metaphorically talk about anger, the bodily state of being hot is mapped into the target of the metaphor (anger) and is embodied during processing this metaphor. The results of these corpus studies suggest that this phenomenon takes place across many languages. In other words, the metonymic relationship between base and target of a metonymy-based metaphor means that the same bodily states that are activated with the base of a metaphor are also experienced when that metaphor is used. In fact, a universal metaphor is a single metaphor that is realized in various forms across a variety of languages. When these metaphors are used, the base of the metaphor and behavioral responses that are associated with the target (for example, heat as a behavioral response to anger) are activated or embodied. In this way, the base of the metaphor and some experiences associated with it are simulated during metaphor processing.

## 5. Degree of Sensorimotor Involvement in the Processing of Metaphors

The results of the research reviewed in this paper have suggested that sensorimotor systems play an important role in the processing of metaphors. A question that is raised here is the extent of importance of sensorimotor systems in the processing of various metaphors. One may argue that the role of sensorimotor systems may not be at the same level in the processing of all types of metaphors; processing some metaphors may be more reliant on sensorimotor systems than some other types of metaphor. This seems to be a valid question, as results of a series of studies have shown that various concepts have various degrees of sensorimotor and action effector strength in various modalities, e.g., [88,89,90,91,92,93]. For example, according to the data presented in Lancaster Sensorimotor Norms [91], the concept of *sky* has a strong degree of visual strength (4.7), but it is weak in auditory (0.3), haptic (0.05), gustatory (0.1), and olfactory (0.3) modalities. To take another example, the concept of *shout* has a high degree of auditory strength (4.1), while it is weak in visual (1.3), haptic (0.2), gustatory (0.4), and olfactory (0) modalities. The same is the case with degrees of action effector strengths of concepts. According to the data of Lancaster Sensorimotor Norms [91], the concept of *run* has a strong foot action effector (4.9), but it has a much smaller degree of head action effector strength (2.4). Based on the results of studies that have measured sensorimotor and action effector strength of concepts, it can be suggested that degrees of involvement of sensorimotor systems in the processing of various metaphors are different. When the base of a metaphor is strong in a certain modality (e.g., visual modality), that modality plays a more important role than other modalities in the processing of that metaphor. For example, when an abstract concept (as the target of a metaphor) is metaphorically described in terms of a strongly visual concept (as the base of the metaphor), the visual system plays the most important role in the processing of that metaphor. Importantly, even visual concepts may have various degrees of visual strength. This means that the degree of visual system involvement in the processing of some visual concepts can be stronger for some visual concepts than other visual concepts. For example, the two concepts of *sun* and *airplane* have strong degrees of visual strength (4.7 and 4.1, respectively), but the degree of visual strength of *sun* is higher than the degree of visual strength of *airplane*. Therefore, it can be suggested that the visual system plays a more important role in the processing of a metaphor that has *sun* as its base than a metaphor that has *airplane* as its base. In other words, the degree of sensorimotor involvement in the processing of a metaphor depends on the sensorimotor strength of its base in various modalities.

Another question that may be raised is how a metaphor that has a base with low degrees of sensorimotor strength in all sensory modalities is processed. If strong perceptual strength of the base of a metaphor is the primary medium for understanding the target in terms of the base, it can be concluded that a metaphor that has a base with low sensorimotor strength is processed by another mechanism. To answer this question, it must be noted that, as Lakoff and Johnson [1] argue, the essence of metaphor is to understand one concept (often an abstract one) in terms of another concept (often a concrete one). This means that bases of metaphors often have strong perceptual strengths. High perceptual strength of the base of a metaphor functions as a medium to understand the abstract target through the activation of the sensorimotor system. If the base of the metaphor is very strong in perceptual modalities, this process can be accomplished easily through the activation of sensorimotor systems. If the base is not strong in perceptual modalities, some amodal resources may come into play to process the metaphor.

## 6. Conclusions and Future Directions

In this paper, we reviewed various types of evidence that support metaphorical embodiment. Behavioral studies have shown that metaphoric actions are mentally simulated, and this simulation can be realized in some parts of the body in the form of body movements. Among the studies that were reviewed, almost all of them have investigated the realizations of these simulations in hand gestures or eye movements. However, it is important to note that these simulations can occur in other parts of the body, but the forms of simulation in various parts of the body may be different. For example, an upward metaphorical movement of hand is different from an upward metaphorical movement of shoulder, head, or eyebrow. All of them can be the concrete realizations of a mental simulation, although there are some differences between them. Hand and eye movements during metaphor processing have been the focus of most studies in this category, but it is important to note that there is nothing special about hand and eye movements. Bodily simulation of a metaphoric action may occur in any part of the body. Perhaps one of the key differences between simulations in various parts of the body is the degree of noticeability. Simulation of metaphoric actions are more noticeable and easier to measure in hand and eye movements than in other parts of the body.

Another important point about behavioral studies is that they have primarily focused on directions (upward, downward, etc.) and traces of hand movements, but these two features are not the whole story about a simulation. There are some other aspects in a mental simulation that are not captured by just studying the directions and traces of hand movements. For example, the degree of force and muscle intensification is a feature of a bodily simulation that cannot be captured by just studying directions and traces of body movements. Perspective of a mental simulation is also another aspect that has not been at the focus of attention in studies on mental simulation of metaphoric actions. These aspects of simulation could be as important as directions and traces of movements. Therefore, in future research projects, these almost-ignored aspects of metaphoric action simulation can be examined.

Regarding neuroimaging studies on mental simulations of metaphoric actions, most studies have focused on the activation of arm and leg regions in the motor system. These studies have shown that when an arm (or leg) metaphoric action is processed, arm (or leg) regions of the motor system are activated. Although such observations suggest that metaphoric actions are simulated during processing metaphoric actions, other aspects of mental simulation need to be examined. Both a circling and a straight hand movement are accompanied by activation in the hand areas of the motor system, but there are some differences between the patterns of activation when these two types of movements are produced or mentally simulated. The same is the case with neuroimaging studies on mental imagery. These studies have shown that visual areas are activated during mental imagery and visual recall of past events. However, since an infinite range of visual events can be simulated, there may be an infinite variety in the patters of neural activations in the visual system.

Finally, it should be noted that behavioral, neuroimaging, and corpus studies have provided evidence that supports metaphorical embodiment. However, there are some aspects of cognition that cannot be explained by theories of embodied cognition and metaphorical embodiment. For instance, people who suffer from Mobius syndrome or spinal injuries do not have the ability to move some parts of their bodies. Some of them may have never moved their bodies, but they still have the ability to recognize and imagine body movements. Inability to move lower limbs does not lead to inability in distinguishing between words that refer to lower limb actions. Even congenitally blinded people can understand visual metaphors [94]. Although theories of embodied cognition and metaphorical embodiment have explained many aspects of cognition, they have failed to fully explain such cases [95]. Therefore, further research is needed to modify current theories of embodied cognition and metaphorical embodiment to offer a more comprehensive picture of mental processes involved in metaphor comprehension.

## Data Availability

Not applicable.

## References

[1] Lakoff G., Johnson M. (1980). Metaphors We Live by.

[2] Gibbs R. (1994). The Poetics of Mind: Figurative Thought, Language, and Understanding.

[3] Gallese G., Lakoff G. (2005). The brain’s concepts: The role of the sensory-motor system in conceptual knowledge. Cogn. Neuropsychol..

[4] Lakoff G. (2012). Explaining embodied cognition results. Top. Cogn. Sci..

[5] Lakoff G. (2014). Mapping the brain’s metaphor circuitry: Metaphorical thought in everyday reason. Front. Hum. Neurosci..

[6] Dove G. (2016). Three symbol ungrounding problems: Abstract concepts and the future of embodied cognition. Psychon. Bull. Rev..

[7] Khatin-Zadeh O., Eskandari Z., Cervera-Torres S., Ruiz Fernández S., Farzi R., Marmolejo-Ramos F. (2021). The strong versions of embodied cognition: Three challenges faced. Psychol. Neurosci..

[8] Khatin-Zadeh O., Farsani D., Banaruee H. (2022). A study of the use of iconic and metaphoric gestures with motion-based, static space-based, static object-based and static event-based statements. Behav. Sci..

[9] Khatin-Zadeh O., Farsani D., Hu J., Eskandari Z., Banaruee H. (2023). Gestural embodiment of intensifiers in iconic, metaphoric, and beat Gestures. Behav. Sci..

[10] Khatin-Zadeh O., Farsani D., Hu J., Eskandari Z., Banaruee H. (2023). The impact of manner adverb on the gestural embodiment of actions described by literal and metaphoric sentences. Behav. Sci..

[11] Khatin-Zadeh O., Farsani D., Reali F. (2022). A study of using metaphoric and beat gestures with motion-based and non-motion-based metaphors during retelling stories. Behav. Sci..

[12] Khatin-Zadeh O., Yazdani-Fazlabadi B. (2023). Two mechanisms for understanding mathematical concepts in terms of fictive motions. Mind Brain Educ..

[13] Tirado C., Khatin-Zadeh O., Gastelum M., Jones N.L., Marmolejo-Ramos F. (2018). The strength of weak embodiment. Int. J. Psychol. Res..

[14] Diefenbach C., Rieger M., Massen C., Prinz W. (2013). Action-sentence compatibility: The role of action effects and timing. Front. Psychol..

[15] Fischer M.H., Zwaan R.A. (2008). Embodied language: A review of the role of the motor system in language comprehension. Q. J. Exp. Psychol..

[16] Glenberg A.M., Sato M., Cattaneo L., Riggio L., Palumbo D., Buccino G. (2008). Processing abstract language modulates motor system activity. Q. J. Exp. Psychol..

[17] Greco A. (2021). Spatial and motor aspects in the “Action-Sentence Compatibility Effect”. Front. Psychol..

[18] Kaschak M.P., Borreggine K.L. (2008). Temporal dynamics of the action-sentence compatibility effect. Q. J. Exp. Psychol..

[19] Taylor L.J., Lev-Ari S., Zwaan R.A. (2008). Inferences about action engage action systems. Brain Lang..

[20] Van Dam W.O., Desai R.H. (2017). Embodied simulations are modulated by sentential perspective. Cogn. Sci..

[21] Winter A., Dudschig C., Miller J., Ulrich R., Kaup B. (2022). The action-sentence compatibility effect (ACE): Meta-analysis of a benchmark finding for embodiment. Acta Psychol..

[22] Glenberg A., Kaschak M. (2002). Grounding language in action. Psychon. Bull. Rev..

[23] Borreggine K.L., Kaschak M.P. (2006). The action-sentence compatibility effect: It’s all in the timing. Cogn. Sci..

[24] Li X., Luo D., Wang C., Xia Y., Jin H. (2022). Motor features of abstract verbs determine their representations in the motor system. Front. Psychol..

[25] Horchak O.V., Giger J.C., Pochwatko G. (2014). Simulation of metaphorical actions and discourse comprehension. Metaphor. Symb..

[26] Altmann G.T.M., Liversedge S.P., Gilchrist I.D., Everling S. (2011). The mediation of eye movements by spoken language. The Oxford Handbook of Eye Movements.

[27] Bergen B.K., Lindsay S., Matlock T., Narayanan S. (2007). Spatial and linguistic aspects of visual imagery in sentence comprehension. Cogn. Sci..

[28] Hartmann M., Mast F.W., Fischer M.H. (2015). Spatial biases during mental arithmetic: Evidence from eye movements on a blank screen. Front. Psychol..

[29] Kaschak M.P., Madden C.J., Therriault D.J., Yaxley R.H., Aveyard M., Blanchard A.A., Zwaan R.A. (2005). Perception of motion affects language processing. Cognition.

[30] Lindsay S., Scheepers C., Kamide Y. (2013). To dash or to dawdle: Verb-associated speed of motion influences eye movements during spoken sentence comprehension. PLoS ONE.

[31] Speed L.J., Vigliocco G. (2014). Eye movements reveal the dynamic simulation of speed in language. Cogn. Sci..

[32] Spivey M.J., Geng J.J. (2001). Oculomotor mechanisms activated by imagery and memory: Eye movements to absent objects. Psychol. Res..

[33] Stocker K. (2014). The Theory of Cognitive Spacetime. Metaphor. Symb..

[34] Stocker K., Hartmann M., Martarelli C.S., Mast F.W. (2016). Eye movements reveal mental looking through time. Cogn. Sci..

[35] Richardson D., Matlock T. (2007). The integration of figurative language and static depictions: An eye movement study of fictive motion. Cognition.

[36] Castaño E., Carrol G. (2020). Mental simulation in the processing of literal and metaphorical motion language: An eye movement study. Metaphor. Symb..

[37] Mishra R.K., Singh N. (2010). Online fictive motion understanding: An eye-movement study with Hindi. Metaphor. Symb..

[38] Singh N., Mishra R.K. (2010). Simulating motion in figurative language comprehension. Open Neuroimaging J..

[39] Wilson N.L., Gibbs R.W. (2007). Real and imagined body movement primes metaphor comprehension. Cogn. Sci..

[40] Khatin-Zadeh O., Hu J., Marmolejo-Ramos F., Farsani D. (2022). The impact of gestural representation of metaphor schema on metaphor comprehension. Pozn. Stud. Contemp. Linguist..

[41] Khatin-Zadeh O. (2023). Embodied metaphor processing: A study of the priming impact of congruent and opposite gestural representations of metaphor schema on metaphor comprehension. Metaphor. Symb..

[42] Alibali M.W., Nathan M.J., Goldman R., Pea R., Barron B., Derry S.J. (2007). Teachers’ gestures as a means of scaffolding students’ understanding: Evidence from an early algebra lesson. Video Research in the Learning Sciences.

[43] Alibali M.W., Nathan M.J. (2012). Embodiment in mathematics teaching and learning: Evidence from learners’ and teachers’ gestures. J. Learn. Sci..

[44] Boroditsky L., Ramscar M. (2002). The roles of body and mind in abstract thought. Psychol. Sci..

[45] Cook S.W., Fenn K.M., Church R.B., Alibali M.W., Kelly S.D. (2017). The function of gesture in learning and memory. Why Gesture? How the Hands Function in Speaking, Thinking and Communicating.

[46] Cook S.W., Yip T.K., Goldin-Meadow S. (2012). Gestures, but not meaningless movements, lighten working memory load when explaining math. Lang. Cogn. Process..

[47] Goldin-Meadow S., Nusbaum H., Kelly S.D., Wagner S. (2001). Explaining math: Gesturing lightens the load. Psychol. Sci..

[48] Khatin-Zadeh O., Eskandari Z., Farsani D. (2023). The roles of mathematical metaphors and gestures in the understanding of abstract mathematical concepts. J. Humanist. Math..

[49] Khatin-Zadeh O., Eskandari Z., Yazdani-Fazlabadi B., Marmolejo-Ramos F. (2022). Four functions of gesture in promoting thought processes. Psychol. Stud..

[50] McNeill D. (1992). Hand and Mind: What Gestures Reveal about Thought.

[51] Pinhas M., Fischer M.H. (2008). Mental movements without magnitude? A study of spatial biases in symbolic arithmetic. Cognition.

[52] Pinhas M., Shaki S., Fischer M.H. (2014). Heed the signs: Operation signs have spatial associations. Q. J. Exp. Psychol..

[53] Masson N., Pesenti M. (2014). Attentional bias induced by solving simple and complex addition and subtraction problems. Q. J. Exp. Psychol..

[54] Cho P.S., So W.C. (2018). A Feel for numbers: The changing role of gesture in manipulating the mental representation of an abacus among children at different skill levels. Front. Psychol..

[55] Hostetter A.B., Alibali M.W. (2008). Visible embodiment: Gestures as simulated action. Psychon. Bull. Rev..

[56] Hostetter A.B., Alibali M.W. (2018). Gesture as simulated action: Revisiting the framework. Psychon. Bull. Rev..

[57] Cacciari C., Bolognini N., Senna I., Pellicciari M.C., Miniussi C., Papagno C. (2011). Literal, fictive and metaphorical motion sentences preserve the motion component of the verb: A TMS study. Brain Lang..

[58] Lai V.T., Howerton O., Desai R.H. (2019). Concrete processing of action metaphors: Evidence from ERP. Brain Res..

[59] Mashal N., Faust M., Hendler T., Jung-Beeman M. (2009). An fMRI study of processing novel metaphoric sentences. Laterality.

[60] Pulvermüller F. (2013). How neurons make meaning: Brain mechanisms for embodied and abstract-symbolic semantics. Trends Cogn. Sci..

[61] Reilly M., Howerton O., Desai R.H. (2019). Time-Course of motor involvement in literal and metaphoric action sentence processing: A TMS study. Front. Psychol..

[62] Romero Lauro L.J., Mattavelli G., Papagno C., Tettamanti M. (2013). She runs, the road runs, my mind runs, bad blood runs between us: Literal and figurative motion verbs: An fMRI study. Neuroimage.

[63] Desai R.H., Binder J.R., Conant L.L., Mano Q.R., Seidenberg M.S. (2011). The neural career of sensory-motor metaphors. J. Cogn. Neurosci..

[64] Johari K., Riccardi N., Malyutina S., Modi M., Desai R.H. (2021). HD-tDCS over motor cortex facilitates figurative and literal action sentence processing. Neuropsychologia.

[65] Desai R.H. (2022). Are metaphors embodied? The neural evidence. Psychol. Res..

[66] Boulenger V., Hauk O., Pulvermüller F. (2009). Grasping ideas with the motor system: Semantic somatotopy in idiom comprehension. Cereb. Cortex.

[67] Boulenger V., Shtyrov Y., Pulvermüller F. (2012). When do you grasp the idea? MEG evidence for instantaneous idiom understanding. Neuroimage.

[68] Desai R.H., Conant L.L., Binder J.R., Park H., Seidenberg M.S. (2013). A piece of the action: Modulation of sensory-motor regions by action idioms and metaphors. Neuroimage.

[69] Yang J., Shu H. (2016). Involvement of the motor system in comprehension of non-literal action language: A meta-analysis study. Brain Topogr..

[70] Bartolomeo P. (2022). The relationship between visual perception and visual mental imagery: A reappraisal of the neuropsychological evidence. Cortex.

[71] Daselaar S.M., Porat Y., Huijbers W., Pennartz C.M. (2010). Modality-specific and modality-independent components of the human imagery system. Neuroimage.

[72] Farah M.J. (1989). The neural basis of mental imagery. Trends Neurosci..

[73] Hamamé C.M., Vidal J.R., Ossandón T., Jerbi K., Dalal S.S., Minotti L., Bertrand O., Kahane P., Lachaux J.P. (2012). Reading the mind’s eye: Online detection of visuo-spatial working memory and visual imagery in the inferior temporal lobe. Neuroimage.

[74] Huijbers W., Pennartz C.M., Rubin D.C., Daselaar S.M. (2011). Imagery and retrieval of auditory and visual information: Neural correlates of successful and unsuccessful performance. Neuropsychologia.

[75] Boccia M., Sulpizio V., Palermo L., Piccardi L., Guariglia C., Galati G. (2017). I can see where you would be: Patterns of fMRI activity reveal imagined landmarks. Neuroimage.

[76] Boccia M., Sulpizio V., Teghil A., Palermo L., Piccardi L., Galati G., Guariglia C. (2019). The dynamic contribution of the high-level visual cortex to imagery and perception. Hum. Brain Mapp..

[77] Zimmer H.D. (2008). Visual and spatial working memory: From boxes to networks. Neurosci. Biobehav. Rev..

[78] Gibbs R.W., Ströbel L. (2016). Experimental and corpus studies on embodied metaphoric meaning. Sensory Motor Concepts in Language and Cognition.

[79] Khatin-Zadeh O., Banaruee H., Reali F., Tirado C., Ruiz-Fernandez S., Yamada Y., Wang R., Nicolas R., Khwaileh T., Szychowska M. (2023). Metaphors of time across cultures. J. Cult. Cogn. Sci..

[80] Johansson-Falck M., Gibbs R.W. (2012). Embodied motivations for metaphoric meaning. Cogn. Linguist..

[81] Kövecses Z. (2005). Metaphor in Culture: Universality and Variation.

[82] Kövecses Z. (2013). The metaphor-metonymy relationship: Correlation metaphors are based on metonymy. Metaphor. Symb..

[83] Kövecses Z. (1986). Metaphors of Anger, Pride, and Love.

[84] Kövecses Z. (1990). Emotion Concepts.

[85] Kövecses Z. (2000). Metaphor and Emotion.

[86] Kövecses Z., Gibbs R. (2008). Metaphor and emotion. The Cambridge Handbook of Metaphor and Thought.

[87] Lakoff G., Kövecses Z., Holland D., Quinn N. (1987). The cognitive model of anger inherent in American English. Cultural Models in Language and Thought.

[88] Chedid G., Brambati S.M., Bedetti C., Rey A.E., Wilson M.A., Vallet G.T. (2019). Visual and auditory perceptual strength norms for 3596 French nouns and their relationship with other psycholinguistic variables. Behav. Res. Method.

[89] Chen I.H., Zhao Q., Long Y., Lu Q., Huang C.R. (2019). Mandarin Chinese modality exclusivity norms. PLoS ONE.

[90] Filipović Đurđević D.F., Popović Stijačić M., Karapandžić J., Halupka-Rešetar S., Martínez-Ferreiro S. (2016). A quest for sources of perceptual richness: Several candidates. Studies in Language and Mind.

[91] Lynott D., Connell L., Brysbaert M., Brand J., Carney J. (2019). The Lancaster Sensorimotor Norms: Multidimensional measures of perceptual and action strength for 40,000 English words. Behav. Res. Method.

[92] Miceli A., Wauthia E., Lefebvre L., Ris L., Simoes Loureiro I. (2021). Perceptual and interoceptive strength norms for 270 French words. Front. Psychol..

[93] Miklashevsky A. (2018). Perceptual experience norms for 506 Russian nouns: Modality rating, spatial localization, manipulability, imageability and other variables. J. Psycholinguist. Res..

[94] Minervino R.A., Martín A., Tavernini L.M., Trench M. (2018). The understanding of visual metaphors by the congenitally blind. Front. Psychol..

[95] Moro V., Scandola M., Aglioti S.M. (2022). What the study of spinal cord injured patients can tell us about the significance of the body in cognition. Psychon. Bull. Rev..

